# Retroperitoneal Approach for Ureteropelvic Junction Obstruction: Encouraging Preliminary Results With Robot-Assisted Laparoscopic Repair

**DOI:** 10.3389/fped.2019.00209

**Published:** 2019-05-28

**Authors:** Thomas Blanc, Jules Kohaut, Caroline Elie, Pauline Clermidi, Luca Pio, Caroline Harte, Enrico Brönnimann, Nathalie Botto, Véronique Rousseau, Pascale Sonigo, Christophe Vaessen, Henri Lottmann, Yves Aigrain

**Affiliations:** ^1^Service de Chirurgie Viscérale et Urologie Pédiatriques, APHP, Hôpital Necker, Paris, France; ^2^Université Sorbonne Paris Cité, Paris, France; ^3^Département Croissance et Signalisation, Hôpital Necker Enfants Malades, Institut Necker Enfants Malades, INSERM U1151-CNRS UMR 8253, Université Paris Descartes, Paris, France; ^4^Unité de Recherche Clinique/Centre d'investigation Clinique Paris Descartes Necker Cochin, Hôpital Universitaire Necker Enfants Malades Paris, Assistance Publique-Hôpitaux de Paris, Paris, France; ^5^Département d'anesthésie-réanimation, APHP, Hôpital Necker, Paris, France; ^6^Radiologie Pédiatrique, APHP, Hôpital Necker, Paris, France; ^7^Service d'urologie, APHP, Hôpital Pitié-Salpétrière, Paris, France

**Keywords:** children, ureteropelvic junction obstruction, pyeloplasty, robotic surgery, retroperitoneal

## Abstract

**Introduction stating the aim of the study:** Robot-assisted laparoscopic pyeloplasty (RALP) is gaining acceptance among pediatric urologists. Few studies have evaluated the retroperitoneal approach for RALP. We share our experience from the first 2 years of a multidisciplinary pediatric robotic program in our center.

**Patients (or Materials) and Methods:** We performed a retrospective analysis of prospectively collected data of children undergoing RALP for ureteropelvic junction obstruction (*n* = 50). Diagnosis was confirmed by ultrasound and Tc-99m mercaptoacetyltriglycine renal scan or MRI; the same criteria were used to evaluate outcome. Surgical approach was chosen according to a specific algorithm. Transperitoneal approach (*n* = 13) was reserved for horseshoe kidney, ectopic kidney, and redo surgery. We analyzed the 37 cases performed by a lateral retroperitoneal approach. Dismembered pyeloplasty was done for all cases and anastomosis was performed using a running monofilament 6/0 absorbable suture. All were drained by double J stent. Patient data, operating room parameters and postoperative course were recorded.

**Results:** The median age was 7.9 years (5.1–13.8); the youngest was 2 years old. The median weight was 23 kg (17–41) with the smallest weighing 12.4 kg. Aberrant crossing vessels were present in 18 children. Median set-up time, from skin incision until the end of the 4-port insertion, was 33 min (29–48). Median surgeon's console time was 151 min (136–182). No conversion to an open procedure was necessary. The postoperative course was free of complications, except urinary tract infection in 6 children. All but 4 patients were discharged on day one. Median follow-up was 9 months (5–13). Redo pyeloplasty was not required. Practical training of other colleagues was possible after 10 cases performed by the same surgeon.

**Conclusion:** These preliminary results suggest that retroperitoneal RALP in children is feasible, safe and effective. It is an excellent option with ideal anatomical exposure. Longer term results as well as continued practice will identify and overcome any challenges and enable surgical mastery of this procedure which is still evolving.

## Introduction

The European Association of Urology Pediatric guidelines acknowledge for pyeloplasty procedure that “in good and experienced hands, the open, laparoscopic, or robotic approaches have the same good outcome” (1). This statement is not based on level 1 evidence and pertains only to pyeloplasty. Thanks to its precise suturing, the robot-assisted approach is used in the majority of minimally invasive pyeloplasty (more than 80%) of teenagers in the United States (2–4).

It remains controversial whether to use a transperitoneal or retroperitoneal approach for pyeloplasty in children.

A large single-center series with long-term follow-up addressed the impact of 10-year retroperitoneal laparoscopic pyeloplasty experience in a pediatric teaching center, demonstrating that it is a safe, reliable, and efficient procedure with an excellent outcome (5). Surgeons from our department have been trained in the retroperitoneal laparoscopic approach. Retroperitoneoscopic pyeloplasty, as described by Yeung et al. has been the standard treatment for ureteropelvic junction obstruction (UPJO) at our institution since 2010 and remained the approach of choice when we started the multidisciplinary pediatric robotic program in our center (6). Publications evaluating the retroperitoneal approach for robot-assisted laparoscopic pyeloplasty are limited (7, 8).

We present our findings in terms of safety and efficacy during the first 2 years of a multidisciplinary pediatric robotic program.

## Materials and Methods

**Figure 1:**
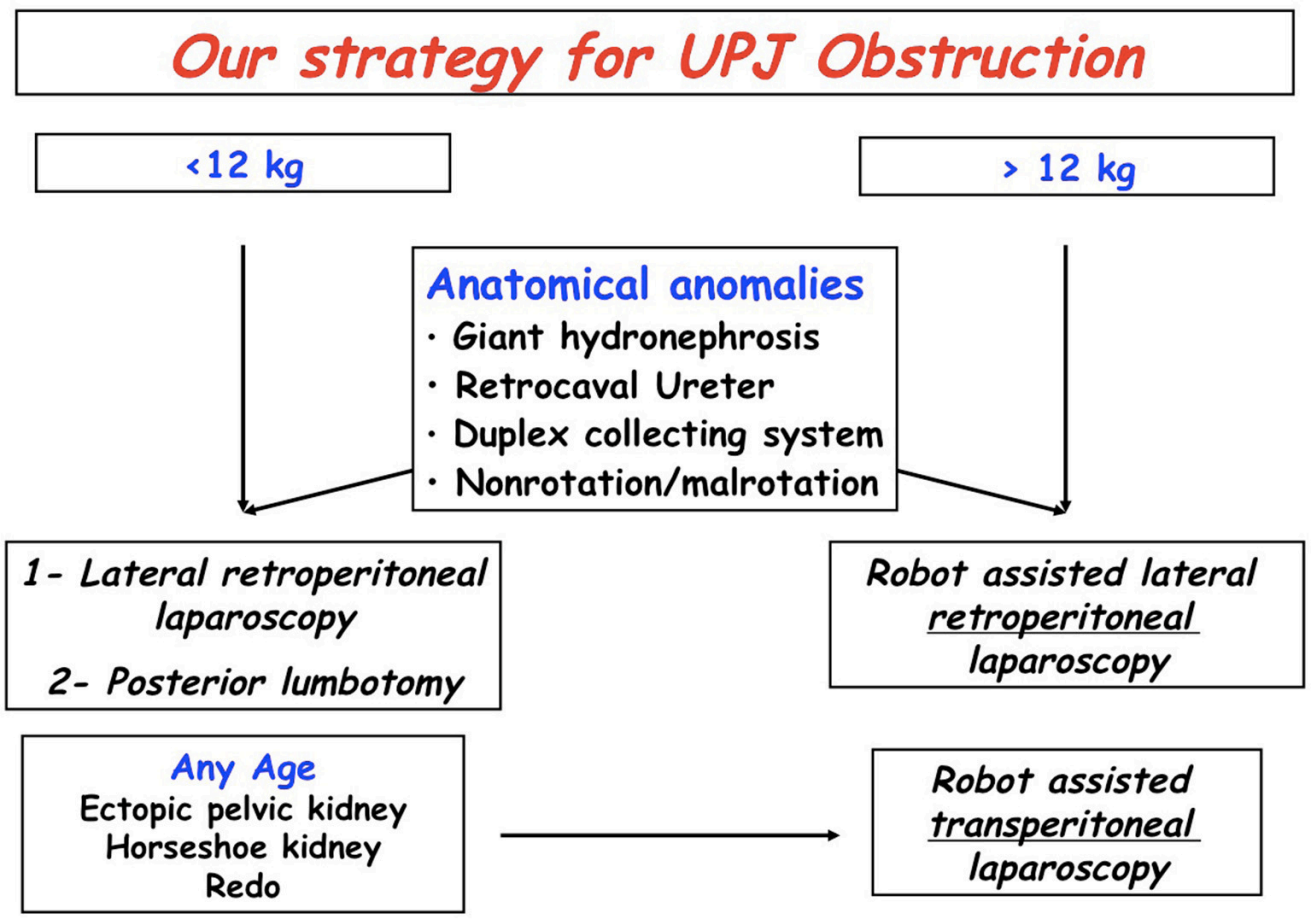
Our strategy for UPJ Obstruction.

Between December 2016 and November 2018, with the Da Vinci Xi Surgical System, we performed 50 robot-assisted laparoscopic pyeloplasties (RALP): 37 by a retroperitoneal approach and 13 by a transperitoneal approach for redo procedure, horseshoe kidney, ectopic kidney, based on a previously published algorithm that we have since modified (Figure 1) (5).

All 50 cases were either done by or with the assistance of one surgeon (TB) who had no prior experience in robotic surgery but who had extensive experience in the retroperitoneal approach (nephrectomy, pyeloplasty). Before starting robotics, the main author had performed almost one hundred laparoscopic retroperitoneal or transperitoneal pyeloplasties (Unpublished data). He also observed an experienced operator in robotics (one of the co-authors Christophe VAESSEN), who later on assisted him with his first procedure.

This retrospective analysis of prospectively collected data received approval from an independent ethics committee (Comité de Protection des Personnes, CPP Ile de France VII). The sponsor was Assistance Publique—Hôpitaux de Paris (APHP, Clinical Research and Innovation Delegation) and this project was funded by a grant from Necker Hospital. It is registered with the ClinicalTrials.gov identifier NCT03274050.

The diagnosis of UPJO was confirmed by renal ultrasound, Uro-magnetic resonance imaging (Uro-MRI) and technetium Tc 99m mercaptoacetyltriglycine-3 (MAG-3) renal scan (RS). Those with equal differential renal function (DRF) on RS were symptomatic (ipsilateral flank pain and/or recurrent febrile urinary tract infections and/or high blood pressure) with pyelocaliceal dilatation on ultrasound.

### Surgical Positioning and Technique

The child is placed in the lateral position close to the edge of the table, with minimum flexion, using lumbar padding to stretch the costo-iliac distance without flexing the operating table. Non-stretch adhesive banding secures this position and prevents displacement either forwards or backwards. The upper leg is stretched while the lower leg is flexed, with no contact between them to avoid compression.

**Figure 2:**
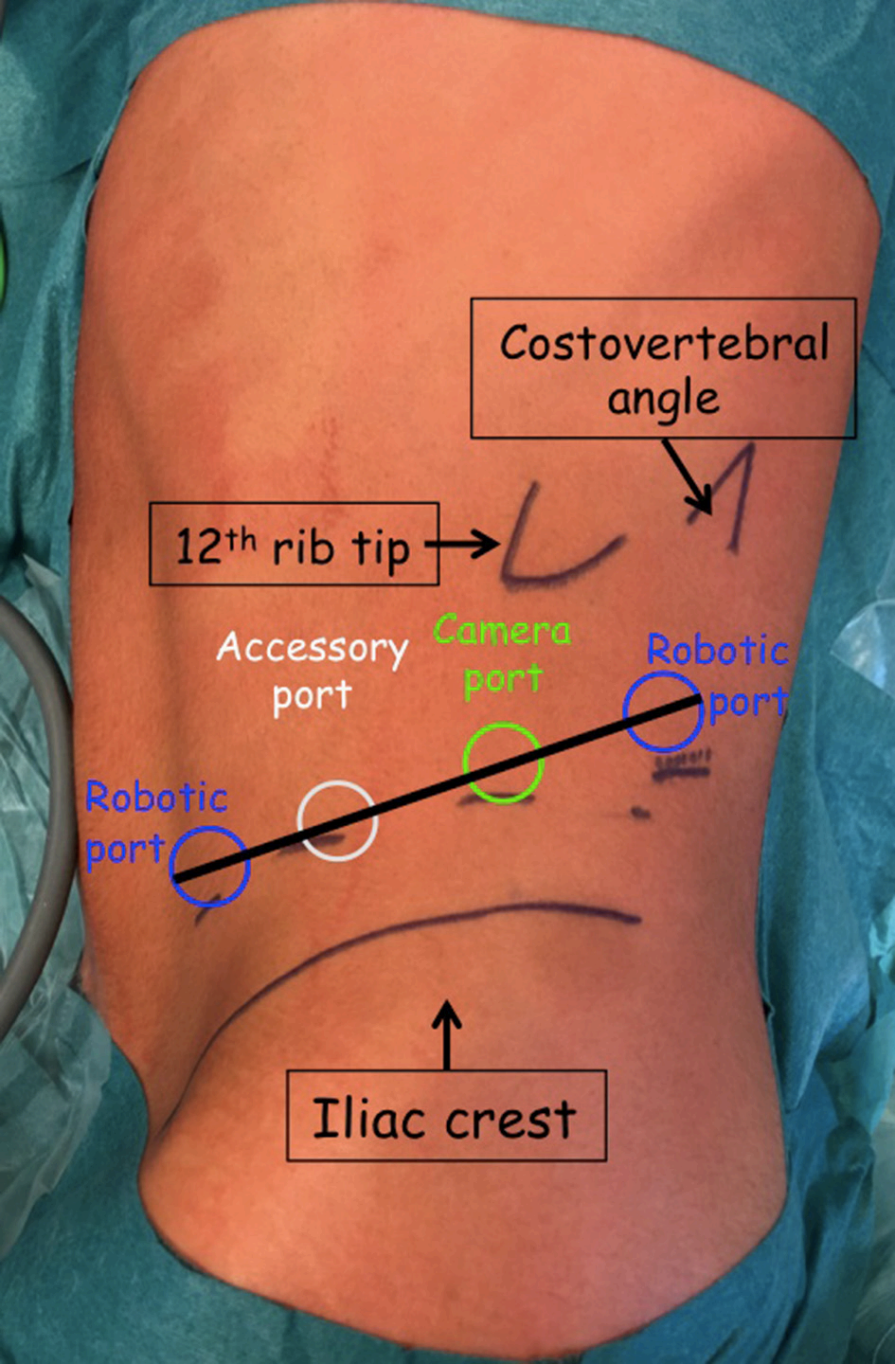
Port placement for left lateral retroperitoneal RAL pyeloplasty.

Port placement is the same for all cases. Three 8-mm robotic ports and one 8-mm AirSeal^®^ iFS System assistant port are placed in an imaginary line drawn from the iliovertebral angle to the iliac fossa (Figure 2).

**Figure 3:**
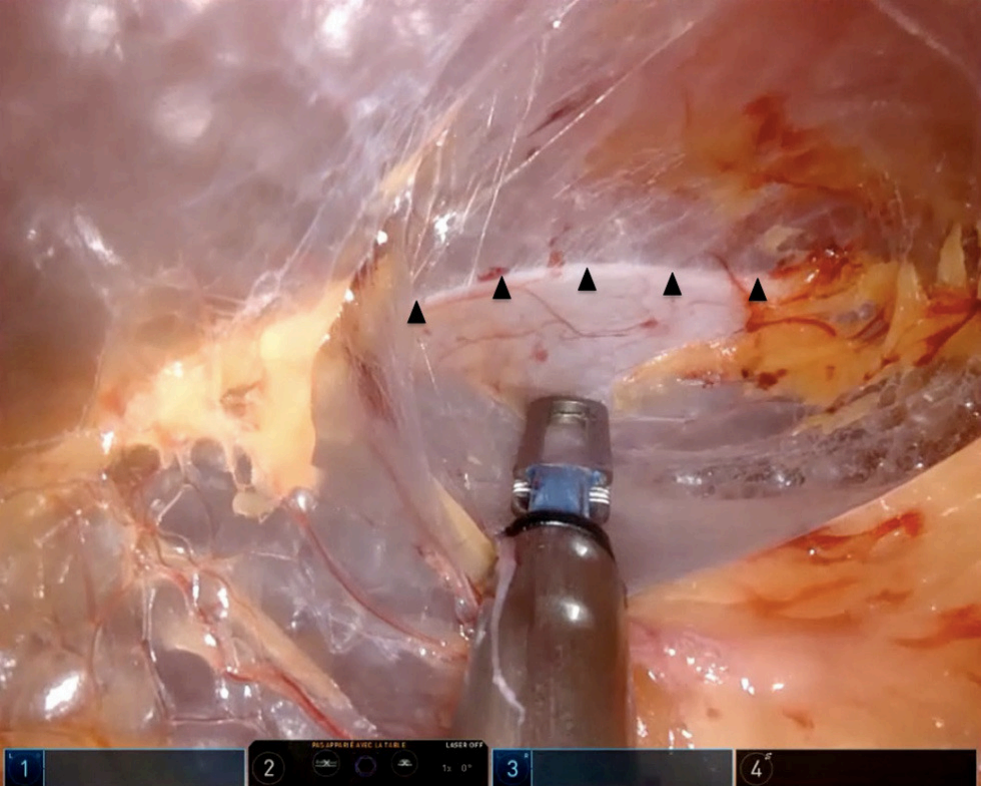
Peritoneal reflection (arrowheads).

- A first 15 mm incision is made in the mid axillary line, at a point between 1/3 and 2/3 extending from the iliac crest to the 12th rib and retroperitoneal access is achieved with a muscle splitting blunt dissection. The first trocar is fixed with a 0 PDS purse-string suture that is applied around the muscles to ensure an airtight seal and to allow traction with the Hasson cone in order to increase the working space. The retroperitoneal space is created with the camera (8-mm; 0°) by blunt dissection and gas insufflation dissection, with no need for finger or balloon dissection.
- The second port is inserted under direct vision at the angle of the iliac crest and the lateral border of the paraspinal muscles.
- To avoid transperitoneal insertion of the 3rd 8-mm robotic port in the iliac fossa, on the edge of the rectus abdominis muscle, the retroperitoneal working space is fully developed by identifying the deep surface of the anterior wall muscles and pushing the peritoneum medially with a laparoscopic bipolar forceps (Figure 3).
- The 8-mm AirSeal^®^ iFS System (ConMed Corporation) assistant port is then inserted in between the camera port and the iliac fossa port. This system is advantageous in providing a stable pneumoperitoneum, constant smoke evacuation and valve-free access.

Insufflation pressure does not exceed 12 mm Hg and the CO_2_ flow rate is 5 liters/min.

The Hasson cone allows for stable and constant traction of the robotic camera port, which is a major advantage with a retroperitoneal limited working space and all the trocars are “burped” as much as possible upwards and outwards, to give an overall 1 to 2 cm space needed for safe maneuvering and to reduce the risk of breaching the peritoneum.

After docking, when the instruments are inserted, the Gerota's fascia is widely opened in a caudo-cranial manner close to the quadratus lumborum muscle with the monopolar curved scissors. Insufflation and gravity push the kidney medially, which correspondingly appears on the upper section of the screen.

Minimal dissection is done to free up the UPJ, and a 4/0 PDS transparietal stay stitch is placed at the junction to limit tissue handling, to give stability and to facilitate suturing. The stay stitch provides variable traction on the ureter and renal pelvis. The ureter is largely spatulated, the UPJ is opened and the pelvis, if required, is resected.

The ureteropelvic anastomosis (Anderson-Hynes pyeloplasty) is initiated with a 6/0 monofilament absorbable running suture with a 3/8-circle needle. After finishing the anterior line of anastomosis, we insert a 4.7F polyurethane double-J stent through the assistant port (either one blind-ended JJ stent or Black-Star^®^ magnetic stent). The Black-Star^®^ (Urotech [Achenmühle, Germany]) is a 4.8 French ureteral stent (length 10–24 cm) with a small magnet fixed with a string at the distal Double-J ureteral stent loop. To remove it, a customized catheter-like retrieval device, lubricated with 2% lidocaine jelly, with a magnetic Tiemann tip is inserted. Both indwelling magnets connect and the catheter can be removed together with the Double-J in an outpatient setting.

The posterior anastomosis is then performed, and the redundant renal pelvis is partially trimmed. No drainage tube is left *in situ* systematically. An indwelling Foley catheter is kept for 24 h. Prophylactic antibiotics are administered by a single dose of ceftriaxone (50 mg/kg) at induction.

In the presence of aberrant polar vessels, the ureter is completely divided and the UPJ and pelvis are delivered anterior to the vessels with the help of the stay stitch. Then the anastomosis is performed as described.

Finally, a plain abdominal film verifies the double-J stent lower end, permitting relocation if it is positioned in the urethra, before waking the patient.

Set-up time is counted from skin incision until the end of the 4-port insertion.

Console time is defined as the time taken to perform the procedure by the surgeon at the master console.

Anastomosis time is the time needed to perform the anterior line of anastomosis, insert the double-J stent and perform the posterior line of anastomosis.

### Complications and Follow Up (FU)

Complications are regarded as any deviation from the expected postoperative course according to the five-grade Clavien classification (9).

Based on our protocol, *FU* consists of a clinical visit associated with renal ultrasound at 1 month after stent removal and then at 6 months, 1, 2, and 5 years (5). *MAG-3* is done in cases of significant asymmetric function in the preoperative study or if FU shows no significant decrease of dilatation on ultrasound or persistence of symptoms (10).

Success is considered objectively as resolution of clinical symptoms, decrease of hydronephrosis on ultrasonography (anteroposterior diameter of renal pelvis, diameter of calices) and improvement of drainage on MAG-3 without further impairment of renal function in patients who had preoperative reduced DRF.

### Statistical Analysis

All statistical analyses were performed using R software (http://cran.r-project.org).

Data were expressed as medians and interquartile ranges (25th, 75th percentiles) for continuous variables, and as numbers and percentages for categorical variables.

Operative time was divided into two categories (< 150 min or ≥150 min). Factors associated with console time were compared between these 2 groups with Chi2 test (or Fisher test if appropriate) and Student *t*-test (or Wilcoxon test if appropriate). *P*-values below 0.05 were considered statistically significant.

The cumulative sum (CUSUM) method was performed to assess the consecutive performances in terms of operative delay over time with reference to pre-defined target (set as the median operative time for all the cases). The CUSUM series was defined as Sn = Σ(Ti–T0), where Ti was the time for an individual i and T0 was the target time.

## Results

Table 1 shows the demographics, indication for surgery, and surgical variables. All cases were completed with the robotic system, with no conversion. We did not experience any robot malfunction, system failure, or complication. No emergency undocking was needed.

**Table 1:** Demographics, indication for surgery and surgical variables, expressed as the medians and interquartile range (25th; 75th percentiles).

	(*N* = 37)
**Age (years)**	7.9 (5.1–13.8)
**Gender**	
Male	19 (51%)
Female	18 (49%)
**Weight (kg)**	23 (17–41)
**Indications for surgery**	
Pain	25 (68%)
UTI	3 (8%)
Pre natal hydronephrosis	5 (13%)
Post natal hydronephrosis	3 (8%)
High blood pressure	1 (3%)
**Side**	
Right	14 (38%)
Left	23 (62%)
**Pre op renal pelvis diameter**	32 (27–39)
**Preop imaging**	
MAG3 renal scan	36 (97%)
Magnetic resonance	9 (25%)
**DRF < 45%**	**22 (61%)**
**Aberrant crossing vessel**	
No	19 (51%)
Yes	18 (49%)
**Stent**	
One blind-ended JJ stent	18 (52%)
Black-Star® magnetic stent	17 (48%)
**Drainage**	2 (5%)
**Set-up time (mins)**	33 (29–48)
**Anastomosis time (mins)**	79 (68–90)
**Console time (mins)**	151 (136–182)
**Conversion**	0
**Hospitalization (days)**	1 (1–1)
**Renal scan postop imaging**	14
Loss of function (<3%)	0
Gain of function (>3%)	9 (64%)
**Follow up (months)**	9 (5–13)
**No. complications**	
Grade I	0
Grade II	8
Grade IIIb	0
**Readmissions**	2
**Second procedure required**	0

The median age was 7.9 years (5.1–13.8); the youngest was 2 years old. The median weight was 23 kg (17–41) with the smallest weighing 12.4 kg. Aberrant crossing vessels were present in 18 children.

There was no significant blood loss.

The peritoneum was opened once during the 3rd port placement, and once during the procedure. To overcome this difficulty, the assistant lifted up the kidney through the assistant port, with no need for conversion to open surgery or laparoscopy

Neither parenchymal injury nor vascular injury in case of polar vessel occurred.

In one patient, preoperative evaluation (ultrasound, MRI, and MAG-3 RS) missed a HSK which was discovered during RALP via the retroperitoneal approach and the procedure was continued using an anterior extraperitoneal approach to the left renal pelvis.

The Black-Star^®^ magnetic stent was inserted in 17 children.

**Figure 4:**
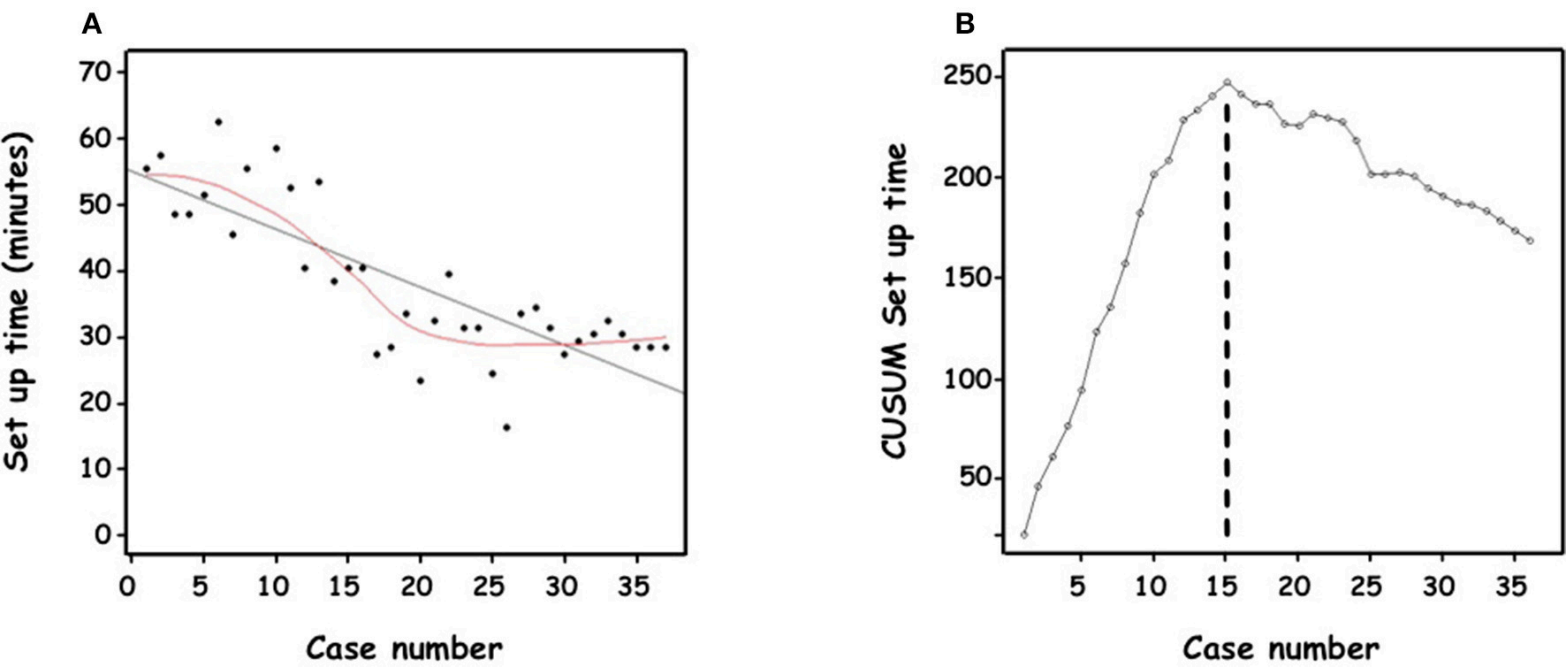
**(A)** Set-up time (minutes) of the consecutive cases of retroperitoneal RAL in chronological order. **(B)** Cumulative sum (CUSUM) chart for set-up time plotted against case number.

Median set-up time, from skin incision until the end of the 4-port insertion, was 33 min (29–48). Initially taking more than an hour, over time the operator became more than twice as proficient (Figure 4A).

The CUSUM methodology demonstrated a biphasic learning curve (Figure 4B). In the first phase, a steep positively sloping curve appeared until the 15th case, indicating the process of overcoming the learning period. It was followed by a flatter negatively sloping curve indicating a period of gaining competence and consolidation without gaining mastery.

**Figure 5:**
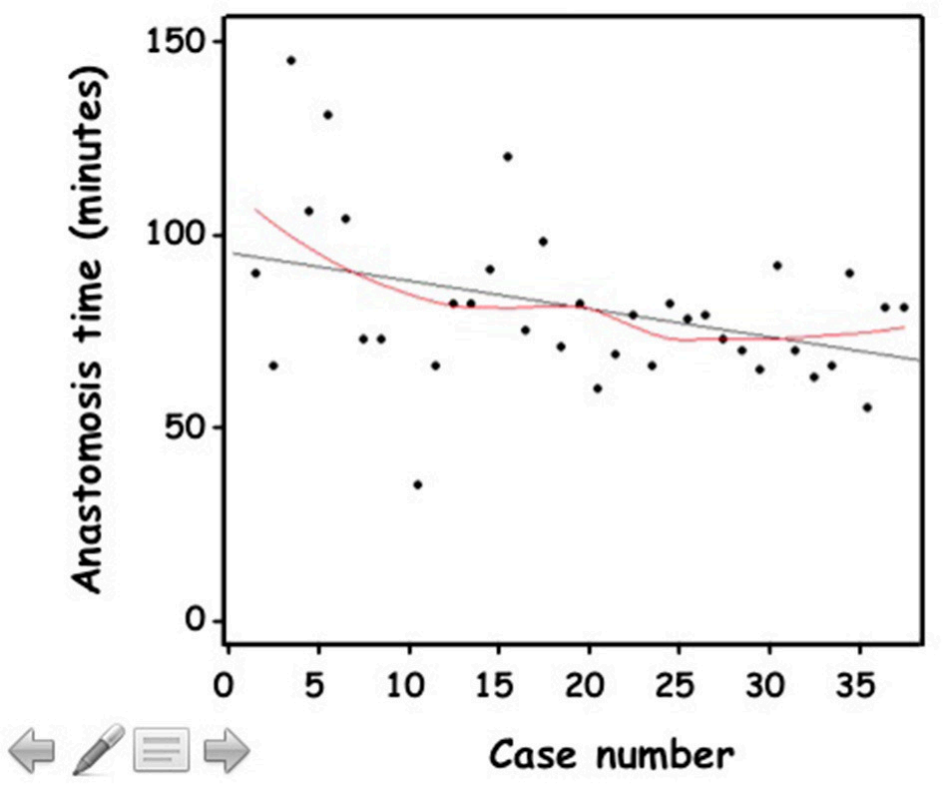
Anastomosis time (minutes) of the consecutive cases of retroperitoneal RAL in chronological order.

**Figure 6:**
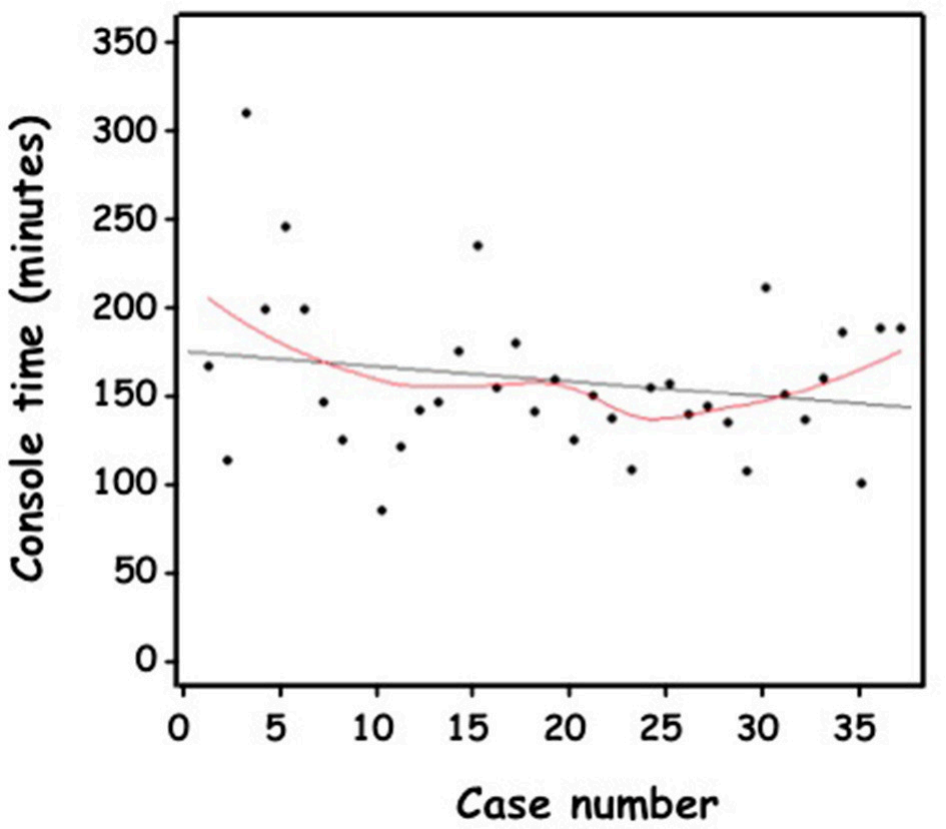
Console time (minutes) of the consecutive cases of retroperitoneal RAL in chronological order.

Median surgeon's console time was 151 min (136–182). For 5 patients, console time was < 120 min; while for 4 patients, console time was >210 min. Median complete anastomosis time was 79 min (68–90). Despite a trend of a flat negatively sloping curve (Figures 5, 6), the CUSUM methodology could not demonstrate 2 phases. Practical training of other colleagues was possible after 10 cases performed by the same surgeon. One senior surgeon and 2 fellows, with extensive experience in laparoscopy, took part in the retroperitoneal RALP operation and were assisted in different steps of the procedure. However, they have not yet performed the procedure independently.

The median hospital stay was 1 day (1–1); all but 4 patients (90%) were discharged the day after the procedure.

The one blind-ended JJ stent was removed after a median of 37 days (31–44) under general anesthesia as a day procedure and the Black-Star^®^ magnetic stent was removed after 34 days (31–35) in the outpatient clinic.

**Figure 7:**
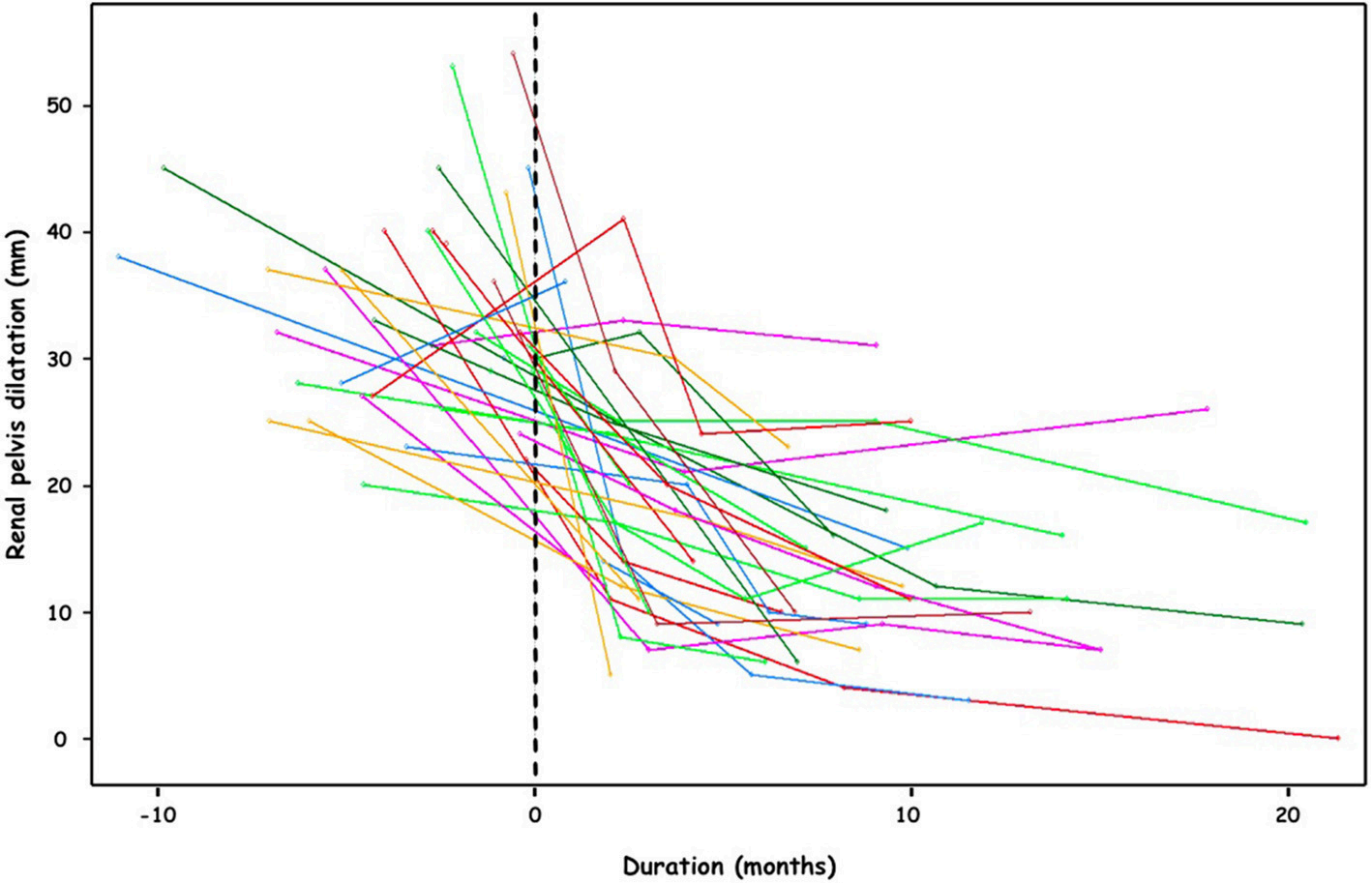
Pre- and post-operative evolution of the renal pelvis dilatation (mm).

All patients have been followed up regularly (median follow-up 9 months (5–13) and remain asymptomatic. All had a decrease of the AP diameter on ultrasound in the postoperative course (Figure 7). Based on our protocol, because of this decrease in AP diameter, we have done post-operative MAG-3 only in cases of asymmetric preoperative function to evaluate the effect of the pyeloplasty on renal function. A total of 14 children have had postoperative MAG-3. No patient has had a decrease in renal function. In 9 children, the operated DRF has improved (>3%). No redo procedure has been necessary.

Six children (4 girls and 2 boys) were treated for 8 febrile UTI in total (Clavien Grade 2) with oral antibiotics. These were not immediate postoperative UTIs, rather they occurred during the period in which the Double-J catheter was indwelling.

Weight was the only significant factor predicting console time [19 kg (16–27) for time < 150 min vs. 31 kg (22–48) for time > = 150 min, *p* = 0.03) (Table 2).

**Table 2:** Factors associated with console time (≥150 min, *n* = 36 surgeries).

**Covariate**	**Console time < 150 min (*N* = 17)**	**Console time ≥150 min (*N* = 19)**	**Univariate analysis *P-*value**
**Age (years)**	5.1 (4.6–8.4)	9.7 (6.3–13.6)	0.11
**Weight (kg)**	19 (16–27)	31 (22–48)	0.03^*^
**Indication for surgery**			
Symptomatic (1)	12 (71%)	15 (79%)	0.71
Asymptomatic (2)	5 (29%)	4 (21%)	
**Aberrant crossing vessel**	7 (41%)	10 (53%)	0.49
**Side**			0.43
Right-sided procedures	5 (29%)	8 (42%)	
Left-sided procedures	12 (71%)	11 (58%)	

*Expressed as the medians and interquartile range (25th; 75th percentiles)*.

*(1) Pain, urinary tract infection*.

*(2) Pre natal hydronephrosis, post natal hydronephrosis, high blood pressure*.

* *Statistically significant*.

## Discussion

The preliminary results of retroperitoneal RALP in children in the debutant 2 years of a multidisciplinary pediatric robotic program in our center demonstrates that this approach can achieve results comparable with those reported with open pyeloplasty, laparoscopy or RALP using the transperitoneal approach (11–13).

There is still some controversy concerning which approach to choose: transperitoneal or retroperitoneal (14).

While the transperitoneal approach offers the advantage of a larger working space, retroperitoneal approach accesses the urinary tract directly and detection of crossing vessels is easier. The potential risk of intra-abdominal organ injury during transperitoneal access, though a rare event, is also avoided. It is a matter of personal preference, based on the experience of the surgeon who should ideally be familiar with both approaches. Since the beginning of the multidisciplinary pediatric robotic program in our center, we have modified the previously published algorithm (Figure 1).

Olsen and Jorgensen published the only series of retroperitoneal RALP cases in 13 pediatric patients (7). Median operative time was 173 min with no obstruction observed at follow-up. Median hospitalization was 2 days and only 1 complication of ureteral stent occlusion occurred. The authors published an expanded series of 65 children with a follow-up of 5 years in 2007, affirming their earlier results (8).

As already highlighted by Olsen et al. previous experience with retroperitoneal pyeloplasties using standard laparoscopic instruments facilitated our transition to this new technology (8). Both approaches share similar basic procedural and technical elements, with the same three instruments being used: monopolar scissors, bipolar forceps, and needle holder. However, there are significant differences related to port placement and size.

For laparoscopic pyeloplasty, we use 3 ports (5-3-3 or 10-5-5 mm) (5). Retroperitoneal access is achieved through the first incision, one finger width from the lower border of the tip of the 12th rib. The Gerota's fascia is opened under direct vision and the first blunt trocar is introduced directly inside the opened Gerota's fascia. A working space is created by gas insufflation dissection. The second trocar is inserted posteriorly in the costovertebral angle, in front of the lumbosacral muscle. Third trocar insertion is in the anterior axillary line, a finger width from the top of the iliac crest.

For robotic pyeloplasty, we have chosen our port placement based on surgical techniques in adult centers and not as described by Olsen et al in 2004 (7). We use 3 8-mm robotic ports and one 8-mm AirSeal^®^ iFS System port, placed linearly from the iliovertebral angle to the iliac fossa, *as already described*. While this accessory (4th) port is optional given that suction/irrigation devices can be passed through the instrument ports of the system in the case of bleeding or need for double-J stent or suture material, it allows us also to operate in greater security and maintain time efficiency. It is very important to keep meticulous hemostasis maintaining a clear vision and thereby orientation in a space with the psoas muscle being a major landmark which is always located on the lower part of the screen.

The robotic trocar diameter is larger (8 vs. 3 or 5 mm) which limits its application to very small children. In our study, the youngest child was 2 years old and 12 kg, older than the infants operated in retroperitoneal laparoscopic pyeloplasty (15). In the future smaller instruments will hopefully be available, making the procedure more suitable for smaller children and infants, as with standard laparoscopic instruments.

However, we feel that instrument size is not the limiting factor as the procedure is performed in a restricted workspace with limited instrument movement, meaning a low risk of collision of the various parts of the robot. The Da Vinci Xi arms are less bulky and therefore much more adapted to pediatric surgery. Thakre et al. have evaluated the performance of robot assisted laparoscopic skills in different workspace sizes with the first generation of the da Vinci Surgical System. While small cubes measuring 40 and 45 mm in diameter have posed difficulties to the surgeon due to arm collisions rendering it impossible to perform drills, these same skills have been replicated with difficulty but no arm contact in cubes measuring 50 and 60 mm, and finally executed with ease in cubes larger than 70 mm, the latter being comparable to the retroperitoneal space in a small child (16).

We suggest that the major limitation lies more in the length/depth needed to operate the robotic instrument in the restricted area that is the retroperitoneal space in small children.

The robotic system mainly speeds up performance of the anastomosis. The LC in robotic surgery is increasingly being analyzed though, for the moment, operative time has been the single parameter used to assess proficiency in studies. Given the disparities within this measure, a case load ranging from 15 to 58 has been thus far proposed as the LC target (17, 18).

We have demonstrated a significant learning curve for the set-up time and a moderate but insignificant learning curve for anastomosis and console times.

In essence, OT is not a reliable measure of learning, and OT alone may not be a useful indicator of good practice (19). Kassite et al. have evaluated the learning curve (LC) over a 10 year period for RALP in children by adopting a multidimensional approach and accounting for patient complexity factors (20). Using the cumulative sum (CUSUM) methodology analyzing multi-outcome parameter, they showed that there were three separate phases for the LC in RALP. Initially, the surgeon was in a learning phase which was followed by a consolidation period and finally progression to increased competence, whereby a significantly shorter operation with reduced hospital stay and less postoperative pain was demonstrated with increased surgical experience.

The role of comfort should not be underestimated in lengthy complex laparoscopic procedures. Pediatric laparoscopic surgeons, who work in very bad ergonomic environments due to the small size of their patients, may have a further increase in static postural stress (21). In 2013, a multicenter survey confirmed a strong association between work-related musculoskeletal symptoms (mainly shoulder symptoms) and the number of laparoscopic procedures performed. Skilled laparoscopic surgeons had more pain than less skilled laparoscopic surgeons. These symptoms were more frequent after laparoscopy than after open procedures. Approximately one in four (27%) of these pediatric laparoscopic surgeons didn't sleep well-due to pain which could potentially hinder surgical performance (22). With the robot, the surgeon is in a more comfortable working position.

## Conclusion

Retroperitoneal RALP in children is feasible, safe and effective. Developing competence and ease with bulky instruments poses a significant learning curve on the surgeon. Longer term follow up as well as continued practice will allow surgical mastery and address any challenges associated with this procedure which is still in its early days.

## Data Availability

The raw data supporting the conclusions of this manuscript will be made available by the authors, without undue reservation, to any qualified researcher.

## Ethics Statement

This retrospective analysis of prospectively collected data received approval from an independent ethics committee (Comité de Protection des Personnes, CPP Ile de France VII). ClinicalTrials.gov identifier NCT03274050.

## Author Contributions

All authors listed have made a substantial, direct and intellectual contribution to the work, and approved it for publication.

### Conflict of Interest Statement

The authors declare that the research was conducted in the absence of any commercial or financial relationships that could be construed as a potential conflict of interest.
